# Calculation of Mercury’s Effects on Neurodevelopment: Bellinger Responds

**DOI:** 10.1289/ehp.1206033R

**Published:** 2012-12-03

**Authors:** David C. Bellinger

**Affiliations:** Harvard Medical School, Harvard School of Public Health, Boston Children’s Hospital, E-mail: david.bellinger@childrens.harvard.edu

In my paper (Bellinger 2012), I noted among the limitations that the calculations are only as valid as the data on which they are based. My hope was that those with a special interest in a particular risk factor would be stimulated to provide stronger data on either the exposure distribution or the dose–response relationship so that the calculations could be refined. I am therefore grateful to Grandjean et al. for providing an updated estimate of the dose–response relationship for pre-natal methyl-mercury, the use of which suggests that the total Full-Scale IQ loss among U.S. children is considerably larger than my initial estimate. All of the estimates listed in Table 2 of my paper (Bellinger 2012) should be considered provisional and should be updated when more precise data become available.
